# Differently Pre-treated Alfalfa Silages Affect the *in vitro* Ruminal Microbiota Composition

**DOI:** 10.3389/fmicb.2019.02761

**Published:** 2019-12-03

**Authors:** Thomas Hartinger, Joan E. Edwards, Ruth Gómez Expósito, Hauke Smidt, Cajo J. F. ter Braak, Nina Gresner, Karl-Heinz Südekum

**Affiliations:** ^1^Institute of Animal Sciences, University of Bonn, Bonn, Germany; ^2^Laboratory of Microbiology, Wageningen University and Research, Wageningen, Netherlands; ^3^Biometrics, Wageningen University and Research, Wageningen, Netherlands

**Keywords:** lucerne silage, rumen microbiota, 16S rRNA gene sequencing, qPCR, Rusitec, bacteria, archaea, anaerobic fungi

## Abstract

Alfalfa (*Medicago sativa* L.) silage (AS) is an important feedstuff in ruminant nutrition. However, its high non-protein nitrogen content often leads to poor ruminal nitrogen retention. Various pre-ensiling treatments differing with respect to dry matter concentrations, wilting intensities and sucrose addition have been previously shown to improve the quality and true protein preservation of AS, and have substantial effects on *in vitro* ruminal fermentation of the resulting silages. However, it is unknown how these pre-ensiling treatments affect the ruminal microbiota composition, and whether alterations in the microbiota explain previously observed differences in ruminal fermentation. Therefore, during AS incubation in a rumen simulation system, liquid and solid phases were sampled 2 and 7 days after first incubating AS, representing an early (ET) and late (LT) time point, respectively. Subsequently, DNA was extracted and qPCR (bacteria, archaea, and anaerobic fungi) and prokaryotic 16S rRNA gene amplicon sequence analyses were performed. At the ET, high dry matter concentration and sucrose addition increased concentrations of archaea in the liquid phase (*P* = 0.001) and anaerobic fungi in the solid phase (*P* < 0.001). At the LT, only sucrose addition increased archaeal concentration in the liquid phase (*P* = 0.014) and anaerobic fungal concentration in the solid phase (*P* < 0.001). Bacterial concentrations were not affected by pre-ensiling treatments. The prokaryotic phylogenetic diversity index decreased in the liquid phase from ET to LT (*P* = 0.034), whereas the solid phase was not affected (*P* = 0.060). This is suggestive of a general adaption of the microbiota to the soluble metabolites released from the incubated AS, particularly regarding the sucrose-treated AS. Redundancy analysis of the sequence data at the genus level indicated that sucrose addition (*P* = 0.001), time point (*P* = 0.001), and their interaction (*P* = 0.001) affected microbial community composition in both phases. In summary, of the pre-ensiling treatments tested sucrose addition had the largest effect on the microbiota, and together with sampling time point affected microbiota composition in both phases of the rumen simulation system. Thus, microbiota composition analysis helped to understand the ruminal fermentation patterns, but could not fully explain them.

## Introduction

Reducing the import of protein-rich feedstuffs, such as soybean meal, in favor of increasing the production of inexpensive on-farm produced protein, such as forage legumes, supports the sustainability of ruminant livestock production (Lüscher et al., 2014), provided that the dietary nitrogen (N) is efficiently utilized in the rumen. Alfalfa (*Medicago sativa* L.) silages (AS) represent an important on-farm grown protein feedstuff for dairy cow rations and are well accepted by ruminants as a dietary component (Dewhurst et al., 2003). Therefore, AS feeding can provide the animals with sufficient amounts of crude protein and dietary fiber (Dewhurst et al., 2003; Coblentz and Grabber, 2013) to maintain rumination activity and microbial protein production in the rumen. However, the bulk of crude protein in AS is in the form of non-protein N (NPN), which is rapidly metabolized to ammonia in the rumen. Excessive ruminal ammonia production results in its absorption by the host, before it is excreted as urea into the environment (Coblentz and Grabber, 2013). In order to improve the ruminal fermentation and N retention, the effects of different alfalfa pre-ensiling treatments, i.e., dry matter (DM) concentration, wilting intensity and sucrose addition on the resulting AS crude protein fractions was investigated using eight different AS (Hartinger et al., 2019a). It was observed that wilting alfalfa with high intensity to 35% DM concentration in combination with sucrose addition at ensiling lead to highest true protein preservation, as well as a generally improved silage quality.

Incubation of the same eight AS in an *in vitro* rumen-simulation system (Rusitec) revealed substantial effects on the ruminal fermentation and fiber degradation as comprehensively discussed in Hartinger et al. (2019b). Mainly, for sucrose-treated AS, increased daily gas production and concentrations of total volatile fatty acids and in specific propionate, n-butyrate, and isovalerate were observed, along with decreased acid detergent fiber degradation (Supplementary Table S1). The DM concentration of the AS showed substantial effects on the ruminal fermentation pattern, with higher gas production and increased concentrations of propionate and isovalerate, as well as lower degradability of neutral and acid detergent fiber. In contrast, high-intensity wilting only enhanced degradation of neutral detergent fiber. These effects of pre-ensiling treatments on ruminal fermentation patterns and fiber degradation of the AS indicate that the microbiota in the Rusitec may have been affected. However, the effect of the different AS on the microbiota has not been investigated so far, but should be since the microbiota can substantially affect animal performance (Guan et al., 2008); an aspect of animal nutrition that has gained more attention during the last decades. Among multiple influencing factors, diet is considered to have the strongest impact on the ruminal microbiota (Henderson et al., 2015). Despite this awareness, microbiota-based research has only focused on the effects of different ensiled forages on the ruminal microbiota (Zhang et al., 2014; Liu et al., 2016), but not on the effect of different pre-ensiling treatments that are commonly applied during forage conservation.

Due to the reduced acid detergent fiber degradability as well as higher gas and volatile fatty acid concentrations during incubation of sucrose-treated AS, we hypothesized contrasting microbiota compositions with higher microbial abundances and diversity in the communities deriving from sucrose-treated AS incubation. Limited influence of the other pre-ensiling treatments on microbiota composition was expected. Secondly, we hypothesized that the microbial community composition changed with prolonged AS incubation, as several fermentation characteristics were previously shown to be altered between early (2 days) and late (7 days) sampling time points, e.g., rise of pH, ammonia-N and isovalerate concentrations. Therefore, the aim of the present study was to assess the effect of the pre-ensiling treatments and incubation time on the microbial community composition and concentrations using liquid and solid phase samples preserved from the previously reported Rusitec study (Hartinger et al., 2019b). This analysis was done using barcoded amplicon sequencing of the prokaryotic 16S rRNA gene, and qPCR analyses of bacteria, archaea, and anaerobic fungi.

## Materials and Methods

### Experimental Design and Sample Collection

An *in vitro* study was previously performed where eight varyingly pre-treated AS differing in terms of crude protein composition and silage fermentation quality (Hartinger et al., 2019a) were incubated in an *in vitro* Rusitec system (Hartinger et al., 2019b). The preparation and chemical composition of the eight AS have been described in detail and can be obtained from Hartinger et al. (2019a). Briefly, pure alfalfa sward was harvested and similarly spread on either black plastic in the sun, i.e., high-intensity wilting treatment (HI), or on white plastic in the shade, i.e., low-intensity wilting treatment (LI). Each of these wilting treatments was used to generate material with 25 or 35% DM concentration (i.e., 25 or 35). These four different sets of treated alfalfa were then ensiled with or without the addition of 125 g/kg DM sucrose (SA), generating a total of eight different AS, which are referred to as: 25HISA, 25HI, 25LISA, 25LI, 35HISA, 35HI, 35LISA, and 35LI.

The eight different AS were subsequently incubated in a Rusitec system for 9 days (Czerkawski and Breckenridge, 1977), the procedure of which has been previously described in detail (Hartinger et al., 2019b). Briefly, each experimental run lasted 17 days, and prior to the incubation of the AS, the Rusitec system was equilibrated by supplementation of a hay and concentrate diet (70:30) for the first 8 days. Subsequently, all silages were incubated isonitrogenously for 9 days. During the AS incubation, two time points were selected, an early time point (ET), i.e., 2 days after the start of the AS incubation, and a late time point (LT), i.e., 7 days after the start of the AS incubation. To obtain the liquid-associated microorganisms, for each time point, vessel fluid (2 ml) was directly collected 2, 4, 12, and 23 h after feed bag exchange. For the solid-associated microorganisms, 48 h feed residues were collected from the vessels 3 and 8 days after the start of AS incubation period as these feed residues were left from the AS fermentation of the prior day, when the liquid phase samples were collected. Subsequently, DM concentration was determined in additional feed residue aliquots and all samples were stored at −80°C until DNA extraction.

### DNA Extraction

Prior to DNA extraction, the four frozen liquid phase samples (i.e., sampled at 2, 4, 12, and 23 h) from each vessel at each time point (i.e., ET or LT) were thawed at 4°C, pooled, and mixed well. An aliquot of the pooled sample (2 ml) was centrifuged at 800 × *g* and 4°C for 15 min to remove feed particles. The supernatant was transferred to a clean tube, and then centrifuged at 21,000 × *g* and 4°C for 40 min to obtain a microbial pellet. For the solid phase samples, a subsample of the feed residues (approximately 5 g wet weight) from each vessel at each time point (i.e., ET or LT) were ground in liquid nitrogen using a mortar and pestle. DNA extraction of liquid and solid phase samples was performed from the obtained microbial pellets and 250 mg (wet weight) of ground feed residue, respectively, using the First-DNA all-tissue Kit (Gen-IAL GmbH, Troisdorf, Germany) according to the manufacturer’s protocol with some minor adjustments. In brief, a bead-beating step with a Precellys^®^ 24 tissue homogenizer (bertin Instruments, Montigny-le-Bretonneux, France) was used to enhance DNA recovery, and an RNase A treatment (VWR International GmbH, Darmstadt, Germany) was performed in order to remove RNA. The DNA yield and purity was determined using a NanoDrop 8000 spectrophotometer (NanoDrop^®^ Technologies, Thermo Fisher Scientific, Waltham, MA, United States), and DNA integrity was checked using agarose gel electrophoresis. Subsequently, DNA extracts were stored at −20°C until further use.

### qPCR

The absolute quantification of the bacterial and archaeal 16S rRNA genes as well as the anaerobic fungal 5.8S rRNA gene was performed on a CFX384 Real-Time PCR Detection System (Bio-Rad Laboratories, Veenendaal, Netherlands). Details of the amplification conditions and reaction mixtures have been previously described elsewhere (van Lingen et al., 2017). Briefly, all qPCR reactions were performed in triplicate using a 10 μl final reaction volume. The forward and reverse primers 1369F/1492R (Suzuki et al., 2000) and 787F/1059R (Yu et al., 2005) were used in SYBR green-based qPCR assays to quantify bacterial and archaeal 16S rRNA genes, respectively. The Neo qPCR For and Neo qPCR Rev primers were applied in a TaqMan probe-based assay to quantify anaerobic fungi (Edwards et al., 2008). Standard curves (10^2^–10^8^ copies/μl) were prepared from custom synthesized DNA prepared from known sequences of *Ruminococcus albus* (sequence data in ENA under accession number: CP002403.1; bacterial qPCR standard), *Methanobrevibacter millerae* (sequence data in ENA under accession number: CP011266.1; archaeal qPCR standard), and *Neocallimastix* sp. (sequence data in ENA under accession number: GU055516.1; fungal qPCR standard). The copy number of each microbial group was calculated per ml vessel fluid and per g DM feed residue for liquid and solid phase, respectively.

### Prokaryotic 16S rRNA Gene Barcoded Amplicon Sequencing

For microbial composition analysis, barcoded amplicons of the V4 region of the prokaryotic 16S rRNA gene were generated using the modified F515-806R primer set (Walters et al., 2016). The PCRs were performed in triplicate with a SensoQuest Labcycler (SensoQuest, Göttingen, Germany) in 35 μl reactions containing 7 μl of 5x HF buffer (Finnzymes, Vantaa, Finland), 0.7 μl of dNTPs (10 mM each; Promega, Leiden, Netherlands), 0.35 μl of Phusion Hot start II DNA polymerase (2 U/μl; Finnzymes, Vantaa, Finland), 0.7μl of the barcoded primer mix (100 μM each), 0.7 μl of template DNA (20 ng/μl) and 25.5 μl of PCR-grade water. The cycling conditions consisted of an initial denaturation at 98°C for 30 s, followed by 25 cycles of: 98°C for 10 s, 50°C for 10 s, 72°C for 10 s, and a final extension step at 72°C for 7 min. The size of the PCR products was confirmed by agarose gel electrophoresis and PCR products were then purified with HighPrep^TM^ (MagBio Europe Ltd., Kent, United Kingdom). Concentrations of the purified PCR products were fluorometrically determined using a Qubit in combination with the dsDNA BR Assay Kit (Invitrogen, Carlsbad, CA, United States). The purified PCR products were then mixed in equimolar amounts into pools including synthetic mock communities, i.e., MC3 and MC4 from Ramiro-Garcia et al. (2016), to control for potential technical biases (Ramiro-Garcia et al., 2016). Samples were then sequenced on the Illumina HiSeq platform (GATC-Biotech, Konstanz, Germany, now part of Eurofins Genomics Germany GmbH) and sequencing data was analyzed using the NG-Tax pipeline version 1.0 (Ramiro-Garcia et al., 2016). Operational taxonomic units (OTU) were defined with an open reference approach and taxonomy was assigned using the SILVA 16S rRNA gene reference database version 128 (Quast et al., 2013).

### Statistical Analyses and Visualization

Data of liquid- and solid-associated microorganisms were analyzed separately. Statistical analysis of the Log_10_ transformed qPCR data was performed with MIXED procedure of SAS version 9.4 (SAS Institute Inc., NC, United States) and repeated measures ANOVA due to the repeated sampling from the same vessel (i.e., ET and LT). To test for the effects of DM concentration, wilting intensity, sucrose addition and their interactions during ET and LT, respectively, the mixed model used was:

Y=i⁢j⁢k⁢l⁢mμ+D+iW+jS+kDWi*+jDSi*+kWSj*+kDWi*Sj*+kV+lR+mei⁢j⁢k⁢l⁢m

where Y_*ijklm*_ is the observed response, μ is the overall mean, D_*i*_ is the fixed effect of DM concentration, W_*j*_ is the fixed effect of wilting intensity, S_*k*_ is the fixed effect of sucrose addition, V_*l*_ is the random effect of vessel, R_*m*_ is the random effect of experimental run, and e_*ijklm*_ is the residual error. Furthermore, the effect of the time point on overall quantities of bacteria, archaea, and anaerobic fungi in the liquid and solid phase was analyzed. When interactions were not significant, differences between least squares means were tested with Tukey’s test. The UNIVARIATE procedure was applied to test assumptions of the model by analysis of the residuals.

The prokaryotic 16S rRNA gene sequence data was analyzed in Rstudio 3.5.3 using the packages phyloseq, microbiome, ape, vegan, picante, and ggplot2 (Dixon, 2003; Paradis et al., 2004; McMurdie and Holmes, 2013; Lahti and Shetty, 2017). For estimating the alpha diversity, phylogenetic diversity (PD) index described by Faith (1994) was calculated and checked using the Shapira-Wilk’s normality method with a *P*-value of > 0.05 confirming normal distribution. Subsequently, the PD data was analyzed using a Kruskal-Wallis test. To determine the effect of pre-ensiling treatments as well as incubation time on beta diversity, unweighted and weighted UniFrac distances were used to perform principal co-ordinate analysis (PCoA). Sample groupings in the PCoA were tested for significance by adonis (Anderson, 2001). Constrained partial redundancy analysis (RDA) of the prokaryotic 16S rRNA sequence data was used to assess the relationship between genus-level phylogenetic groupings of the OTUs and incubation time or pre-ensiling treatments. Liquid and solid phase samples were analyzed separately, and the analysis was performed using Canoco 5.11 (Šmilauer and Lepš, 2014). The data was transformed [log(fraction + 0.0001)] and significance of explanatory variables was tested using a Monte Carlo permutation test with a total of 999 permutations using the factors vessel and experimental run as covariates. The significance level was defined at *P* < 0.05 and trends were declared at 0.05 < *P* < 0.10 for all statistical analyses. Regarding the multivariate data, RDA and PCoA analyses each used a single test statistic based on all genera and OTUs, respectively (a pseudo *F*-value), the significance of which is tested by Monte Carlo simulation and therefore did not need a *P*-value adjustment due to multiple testing.

## Results

### qPCR

In both phases, bacteria were the most abundant group being in general at least 100-fold higher in concentration than archaea and anaerobic fungi (Tables 1, 2). Anaerobic fungi were less than archaea in the solid phase, with only traces of anaerobic fungi detected in the liquid phase as the low quantities were below the detection limit of the assay. The effects of pre-ensiling treatments on microbial concentrations at the ET were assessed (Table 1). In the liquid phase, archaeal concentration was increased by higher DM concentration (*P* = 0.001) and were highest for high DM sucrose-treated AS (*P* = 0.044). High DM concentration and sucrose addition tended to increase the concentration of bacteria (*P* = 0.057) and archaea (*P* = 0.061), respectively, in the liquid phase. For the solid phase, sucrose addition increased the concentration of anaerobic fungi (*P* < 0.001), while DM concentration tended to increase the anaerobic fungal concentration (*P* = 0.057). Wilting intensity had no effect on the concentration of any microbial group (Table 1).

**TABLE 1:** Effect of pre-ensiling treatments^a^ on concentrations of liquid- and solid-associated microorganisms at the early time point.

		**Treatment**									***P-*value**						
				
**Phase**	**Group**	**25HISA**	**25HI**	**25LISA**	**25LI**	**35HISA**	**35HI**	**35LISA**	**35LI**	**SEM^b^**	**DM**	**WI**	**SA**	**DM × WI**	**DM × SA**	**WI × SA**	**DM × WI × SA**
Liquid	Bacteria	9.19	9.33	9.33	9.23	9.36	9.22	9.41	9.38	0.03	0.057	0.362	0.448	0.727	0.173	0.801	0.136
Liquid	Archaea	7.13	7.14	7.18	7.10	7.42	7.12	7.38	7.28	0.04	0.001	0.305	0.061	0.401	0.044	0.798	0.038
Liquid	An. fungi^c^	–^d^	–	–	–	–	–	–	–	–	–	–	–	–	–	–	–
Solid	Bacteria	11.41	11.48	11.45	11.38	11.52	11.37	11.40	11.52	0.02	0.658	0.900	0.918	0.691	0.900	0.564	0.070
Solid	Archaea	9.69	9.73	9.68	9.56	9.73	9.58	9.60	9.80	0.03	0.861	0.727	0.861	0.279	0.616	0.477	0.059
Solid	An. fungi	9.07	8.82	9.21	8.56	9.47	8.84	9.25	8.83	0.10	0.057	0.374	< 0.001	0.963	0.629	0.526	0.066

**Values are provided as Log_10_ transformed rRNA gene copies per ml vessel fluid and g dry matter of feed residue for liquid- and solid-associated microorganisms, respectively. ^*a*^Treatments include different: dry matter (DM) concentrations, i.e., 25 or 35; wilting intensities (WI), i.e., low (LI) or high (HI); and sucrose addition (SA). ^*b*^Standard error of the mean. ^*c*^Anerobic fungi. ^*d*^Below the detection limit of the method.**

**TABLE 2:** Effect of pre-ensiling treatments^a^ on concentrations of liquid- and solid-associated microorganisms at the late time point.

		**Treatment**									***P*-value**						
				
**Phase**	**Group**	**25HISA**	**25HI**	**25LISA**	**25LI**	**35HISA**	**35HI**	**35LISA**	**35LI**	**SEM^b^**	**DM**	**WI**	**SA**	**DM × WI**	**DM × SA**	**WI × SA**	**DM × WI × SA**
Liquid	Bacteria	9.33	9.34	9.26	9.35	9.43	9.28	9.27	9.30	0.02	0.968	0.429	0.936	0.749	0.385	0.345	0.719
Liquid	Archaea	7.56	7.31	7.49	7.44	7.68	7.39	7.49	7.31	0.04	0.574	0.651	0.014	0.527	0.532	0.264	0.969
Liquid	An. fungi^c^	–^d^	–	–	–	–	–	–	–	–	–	–	–	–	–	–	–
Solid	Bacteria	11.68	11.61	11.54	11.69	11.69	11.60	11.48	11.59	0.03	0.253	0.246	0.262	0.487	0.741	0.094	0.808
Solid	Archaea	10.04	10.01	9.86	10.06	9.98	10.02	9.85	9.97	0.03	0.323	0.119	0.050	0.876	0.977	0.136	0.488
Solid	An. fungi	9.47	8.47	8.95	8.41	9.37	8.60	9.29	8.22	0.17	0.738	0.055	< 0.001	0.265	0.418	0.630	0.173

**Values are provided as Log_10_ transformed rRNA gene copies per ml vessel fluid and g dry matter of feed residue for liquid- and solid-associated microorganisms, respectively. ^*a*^Treatments include different: dry matter (DM) concentrations, i.e., 25 or 35; wilting intensities (WI), i.e., low (LI) or high (HI); and sucrose addition (SA). ^*b*^Standard error of the mean. ^*c*^Anerobic fungi. ^*d*^Below the detection limit of the method.**

The effects of pre-ensiling treatments on microbial concentrations at the LT were also assessed (Table 2). Addition of sucrose increased the archaeal concentration in the liquid phase (*P* = 0.014), but tended to decrease archaeal concentration in the solid phase (*P* = 0.050). The anaerobic fungal concentration was increased by sucrose addition (*P* < 0.001), and also tended to be higher during incubation of high-intensity wilted AS (*P* = 0.055). The concentration of bacteria, however, was not affected by any pre-ensiling treatment (Table 2).

The average concentrations of bacteria, archaea, and anaerobic fungi for the eight AS for both phases and time points are summarized in Table 3. Overall, during the transition from the early to the late time point, the concentration of archaea increased for both phases (*P* < 0.001), whereas the bacterial concentration increased only in the solid phase (*P* < 0.001). From the early to the late time point, anaerobic fungi decreased in the solid phase (*P* = 0.036; Table 3).

**TABLE 3:** Effect of time point on concentrations of liquid- and solid-associated microorganisms.

**Phase**	**Group**	**Early**	**Late**	**SEM^a^**	***P*-value**
Liquid	Bacteria	9.30	9.32	0.01	0.743
Liquid	Archaea	7.22	7.46	0.12	<0.001
Liquid	Anaerobic fungi	–^b^	–	–	–
Solid	Bacteria	11.44	11.61	0.08	<0.001
Solid	Archaea	9.67	9.97	0.15	<0.001
Solid	Anaerobic fungi	9.01	8.85	0.08	0.036

**Values are provided as Log_10_ transformed rRNA gene copies per ml vessel fluid and g dry matter of feed residue for liquid- and solid-associated microorganisms, respectively. ^*a*^Standard error of the mean. ^*b*^Below the detection limit of the method.**

### Prokaryotic 16S rRNA Gene Amplicon Sequencing

**FIGURE 1:**
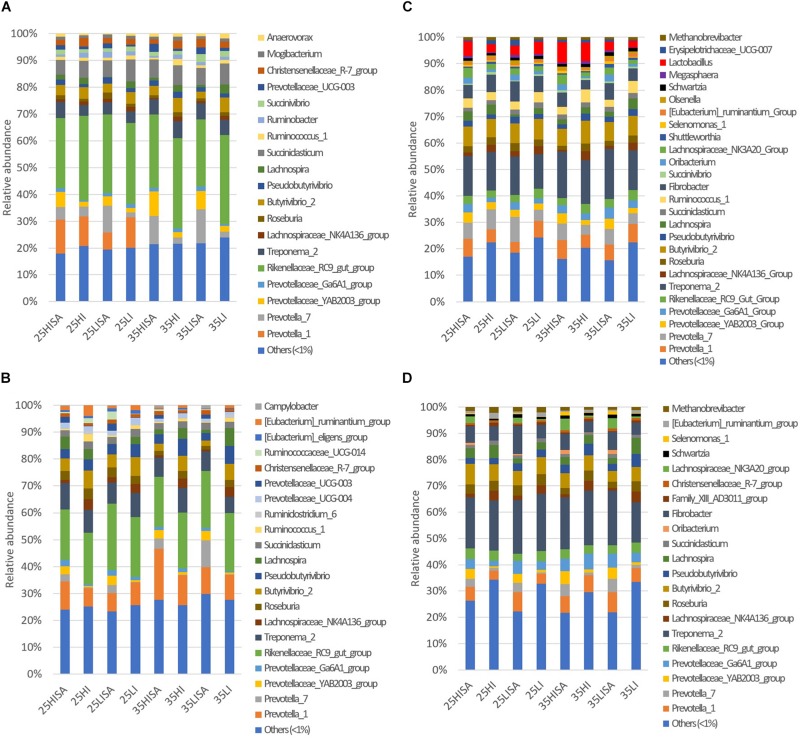
Relative abundances of major prokaryotic genera (>1% relative abundance in at least one alfalfa silage type) and others (<1% relative abundance) in samples for **(A)** liquid phase at the early time point; **(B)** liquid phase at the late time point; **(C)** solid phase at the early time point; **(D)** solid phase at the late time point. Bars represent means (*n* = 4) for each alfalfa silage (AS) type. Abbreviations for each AS type indicate the pre-ensiling treatments including different dry matter concentrations (25 or 35), wilting intensities [low (LI) or high (HI)] and sucrose addition (SA).

The prokaryotic 16S rRNA gene sequence data comprised in total 19,728,379 reads that were assigned to 983 OTUs, of which 822 could be assigned up to genus level (Supplementary Table S2). Analysis of the most abundant (>1%) bacterial and archaeal genera revealed that in the liquid phase, 19 and 21 genera had a relative abundance higher than 1% for the ET and LT, respectively (Figures 1A,B). For the solid phase, 26 and 21 genera were among the most abundant genera at the ET and LT, respectively (Figures 1C,D).

Regarding the liquid phase, *Rikenellaceae*_RC9 gut group was the most abundant genus for both time points, whereas *Treponema*_2 was the most abundant taxon for both time points in the solid phase. The results also showed that the family *Prevotellaceae* was overall highly predominant in both phases at ET and LT, and included the following genera: *Prevotella* 1, *Prevotella 7*, *Prevotellaceae*_Ga6A1 group, and YAB2003 group. Likewise, the bacterial genera *Fibrobacter*, *Butyrivibrio*_2, *Roseburia*, *Pseudobutyrivibrio*, and *Lachnospiraceae* NK4A13 group were predominant in both phases and time points. For the solid phase, OTUs belonging to the archaeal genus *Methanobrevibacter* were also among the most abundant taxa for both time points, whereas no archaeal genus was predominant in the liquid phase.

**FIGURE 2:**
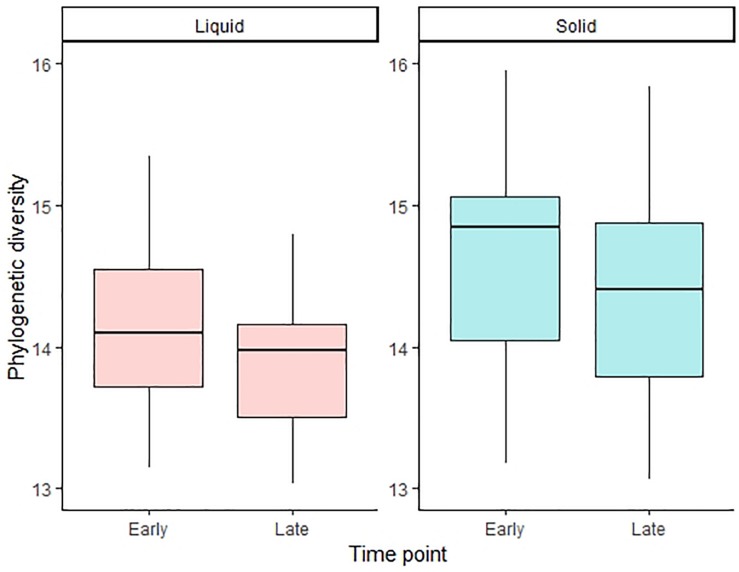
Alpha diversity calculated with the phylogenetic diversity index for the liquid and solid phase and for the early and late time point, respectively. The boxplots show the 25^th^, 50^th^, and 75^th^ percentiles, with whiskers showing the extremes of the data.

Regarding the influences of pre-ensiling treatments on alpha diversity, no effects on the PD index were observed for the ET in both phases (Table 4 and Supplementary Figure S1). For the LT, however, the pre-ensiling treatment sucrose addition increased the PD index (*P* < 0.001) for liquid-associated communities (Table 4). Also, DM concentration × sucrose addition (*P* = 0.002), as well as wilting intensity × sucrose addition (*P* = 0.002) showed higher PD indices for liquid-associated microbes for sucrose-treated AS, irrespective of DM concentration or wilting intensity. For the solid phase at the LT, sucrose addition showed a tendency to increase the PD index (*P* = 0.065; Table 4). Generally, the PD index of liquid-associated microbes decreased from ET to LT (*P* = 0.034; Figure 2). Similarly, the PD index of solid-associated microorganisms tended to be lower during the LT compared to the ET (*P* = 0.060).

**TABLE 4:** Effect of pre-ensiling treatments^a^ on phylogenetic diversity index for liquid-associated and solid-associated microorganisms at early and late time points.

		**Treatment**									***P*-value**						
				
**Phase**	**Time point**	**25HISA**	**25HI**	**25LISA**	**25LI**	**35HISA**	**35HI**	**35LISA**	**35LI**	**SEM^b^**	**DM**	**WI**	**SA**	**DM × WI**	**DM × SA**	**WI × SA**	**DM × WI × SA**
Liquid	Early	14.12	13.96	14.05	13.57	13.86	14.03	14.11	14.30	0.08	0.763	0.547	0.792	0.822	0.853	0.933	0.978
Liquid	Late	14.22	12.73	14.00	13.59	13.93	13.39	14.12	12.81	0.20	0.792	0.763	<0.001	0.790	0.002	0.002	0.010
Solid	Early	14.90	14.63	14.68	14.95	14.71	14.83	14.46	15.05	0.07	0.843	0.906	0.843	0.976	0.740	0.807	0.947
Solid	Late	14.86	14.21	14.34	14.32	14.60	14.11	14.67	14.11	0.10	0.972	0.940	0.065	0.989	0.187	0.345	0.808

**^*a*^Treatments include different: dry matter (DM) concentrations, i.e., 25 or 35; wilting intensities (WI), i.e., low (LI) or high (HI); and sucrose addition (SA). ^*b*^Standard error of the mean.**

**FIGURE 3:**
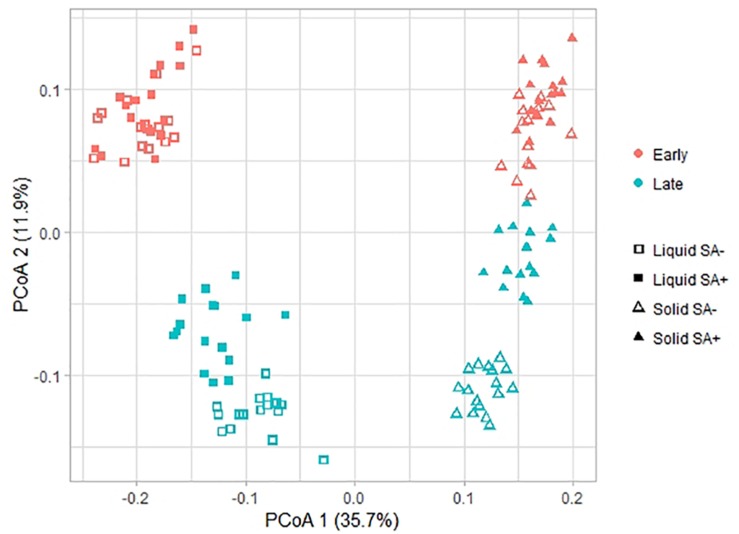
Changes in prokaryotic community composition associated with the time point, phase, and sucrose addition visualized as a principal co-ordinate analysis (PCoA) using weighted UniFrac distance metrics. Symbol shapes indicate the two phases, i.e., liquid and solid, from which the samples originated, colors indicate the different time points, i.e., early and late, and symbol fillings indicate the sucrose addition, i.e., with (SA+) or without (SA−). The percentage of variation explained is indicated on the respective axes.

**FIGURE 4:**
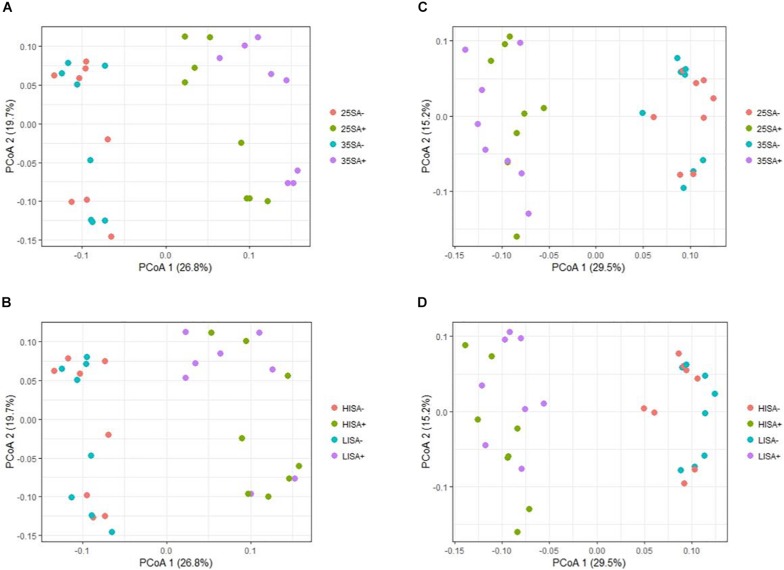
Changes in prokaryotic community composition associated with the interactions of different pre-ensiling treatments at the late time point, visualized as a principal co-ordinate analysis (PCoA) using weighted UniFrac distance metrics for **(A)** treatments dry matter (DM) concentration × sucrose addition in liquid phase samples; **(B)** wilting intensity × sucrose addition in liquid phase samples; **(C)** DM concentration × sucrose addition in solid phase samples; **(D)** and wilting intensity × sucrose addition in solid phase samples. Abbreviations indicate the interactions of treatments including different dry matter concentrations (25 or 35), wilting intensities [low (LI) or high (HI)] and sucrose addition [with (SA+) or without (SA−)]. The percentage of variation explained is indicated on the respective axes.

With respect to beta diversity, PCoA analysis at the OTU-level, using weighted UniFrac distances (Figure 3), displayed a clear separation between the liquid and the solid phase along PCoA axis 1 (*P* = 0.001). Regarding time point, ET samples clustered at the top of PCoA axis 2, while LT samples generally clustered at the bottom (*P* = 0.001). Separation of the ET and LT samples was greater for the liquid than for the solid phase. In the solid phase, sucrose-treated and non-treated samples separated for the LT. The same pattern was observed when using unweighted UniFrac distances (Supplementary Figure S2). For the ET, sucrose addition was the only pre-ensiling treatment that had an effect on beta diversity in the liquid (*P* = 0.005) and solid (*P* = 0.011) phase. Sucrose addition also caused a clear effect on beta diversity at the LT for the liquid (*P* = 0.001) and solid (*P* = 0.001) phase. For the LT, beta diversity was also affected by interactions of sucrose addition × DM concentration (*P* = 0.001) and wilting intensity × sucrose addition (*P* = 0.001), but clear separations were only visible between sucrose-treated and non-sucrose-treated samples along the first PCoA axis (Figure 4). However, for DM concentration × sucrose addition, a weak separation along PCoA axis 1 was found between sucrose-treated low DM AS and sucrose-treated high DM AS (Figure 4A). The same patterns were observed in the corresponding PCoA analysis using unweighted UniFrac distance metrics (Supplementary Figure S3 and Supplementary Table S3).

**FIGURE 5:**
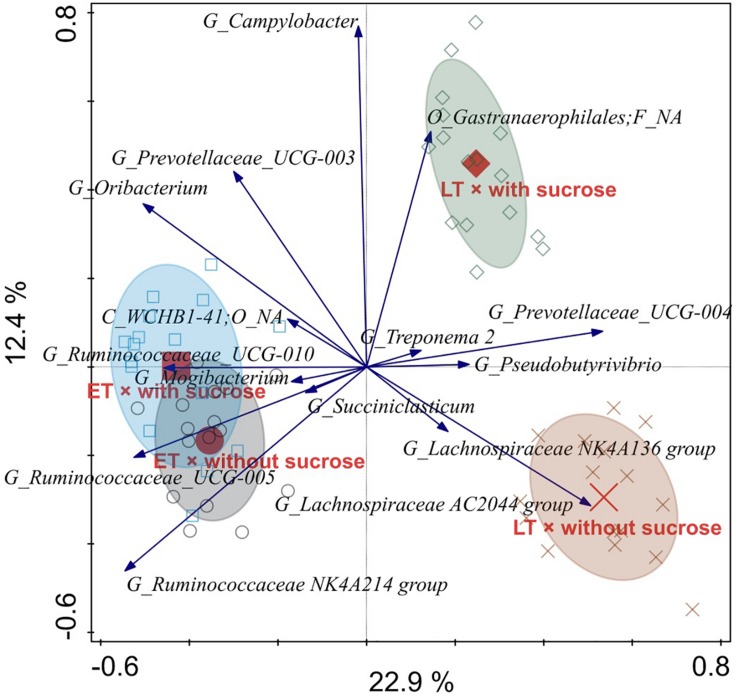
Redundancy analysis triplot illustrating the relationship between the top 15 genus-level phylogenetic groupings of the operational taxonomic units (OTU) for liquid-associated microorganisms explaining the variance of the interaction of time point × sucrose addition with the covariates vessel and experimental run. Arrow length indicates the variance that can be explained by the interactions. Arrow labels indicate the taxonomic identification of genus-level phylogenetic groupings, with the level [i.e., phylum (P), class (C), order (O), family (F), or genus (G)]. Abbreviation NA indicates the levels that could not be annotated, for instance “O_*Gastranaerophilales*; F_NA” was reliably assigned to the order *Gastranaerophilales*, but the family could not be annotated. Sample codes for the means indicate the different time points, i.e., early (ET) and late (LT), as well as the sucrose addition, i.e., with sucrose or without sucrose.

**FIGURE 6:**
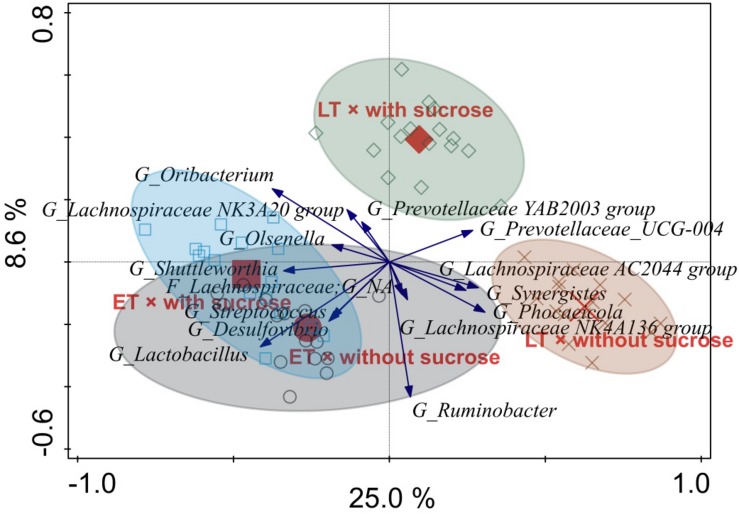
Redundancy analysis triplot illustrating the relationship between the top 15 genus-level phylogenetic groupings of the operational taxonomic units (OTU) for solid-associated microorganisms explaining the variance of the interaction of time point × sucrose addition with the covariates vessel and experimental run. Arrow length indicates the variance that can be explained by the interactions. Arrow labels indicate the taxonomic identification of genus-level phylogenetic groupings, with the level [i.e., phylum (P), class (C), order (O), family (F), or genus (G)]. Abbreviation NA indicates the levels that could not be annotated, for instance “F_*Lachnospiraceae*; G_NA” was reliably assigned to the family *Lachnospiraceae*, but the genus could not be annotated. Sample codes for the means indicate the different time points, i.e., early (ET) and late (LT), as well as the sucrose addition, i.e., with sucrose or without sucrose.

Constrained RDA analysis showed that only sucrose addition (*P* = 0.001) and time point (*P* = 0.001) contributed to explaining the observed variation in the microbial community for both time points in liquid- and solid-associated microorganisms. Time point explained 22.1 and 20.7% of the variation remaining after removal of the between run and between vessel variation for liquid- and solid-associated microorganisms, respectively. Sucrose addition was associated with 10.5 and 11.0% of total variation for liquid- and solid-associated microorganisms, respectively. In addition, the RDA analysis revealed an interaction of time point × sucrose addition for microbes in both phases (both *P* = 0.001), with sucrose addition having a bigger effect at the LT than at the ET (Figures 5, 6).

In the RDA triplot for the liquid phase, samples of different time points separated along the first canonical axis, i.e., from left to right (Figure 5). Irrespective of sucrose addition the following groups belonging to the *Ruminococcaceae* were generally associated with the ET: UCG-010, UCG-005, and NK4A214. A weaker association with the ET was observed for *Mogibacterium* and *Succiniclasticum*. In general, the UCG-004 group belonging to the *Prevotellaceae* was associated with the LT. LT samples clearly separated regarding sucrose addition along the second canonical axis, i.e., from bottom to top. A genus that could only be reliably annotated to the order *Gastranaerophilales* was strongly associated with LT and sucrose addition, whereas the AC2044 group belonging to the *Lachnospiraceae* was strongly associated with LT without sucrose. A weaker association with LT without sucrose was also present for the NK4A136 group belonging to the *Lachnospiraceae*. The NK4A214 group belonging to the *Ruminococcaceae* was positively associated with ET without sucrose.

The RDA triplot for the solid phase showed a similar pattern with samples of different time points separating along the first canonical axis, i.e., from left to right, as well as LT samples separating for sucrose addition along the second canonical axis, i.e., from bottom to top (Figure 6). The genera *Shuttleworthia* and *Lactobacillus* were positively associated with ET. The AC2044 group belonging to the *Lachnospiraceae*, *Synergistes* and *Phocaeicola* were associated with LT without sucrose, and no taxon was associated with LT with sucrose.

Among the most affected genera indicated in the liquid and solid phase RDA plots, respectively, two genera were clearly affected the same way in both phases. These two genera were *Prevotellaceae* UCG-004 (associated with LT) and the AC2044 group belonging to the *Lachnospiraceae* (associated with LT without sucrose).

## Discussion

### Absolute Abundances of Bacteria, Archaea, and Anaerobic Fungi

The qPCR results revealed bacteria to be the most abundant microbial group in both liquid and solid phase, followed by archaea and then anaerobic fungi. This is in line with proportions typically found in the rumen (Puniya et al., 2015; Vaidya et al., 2018). Compared to other Rusitec-based experiments (Martínez et al., 2010; Deckardt et al., 2015; Khiaosa-ard et al., 2015; Wetzels et al., 2018), the abundances of total bacteria and archaea observed in our study were slightly higher, but within a realistic range (Puniya et al., 2015; van Lingen et al., 2017; Vaidya et al., 2018).

Regarding anaerobic fungi, Deckardt et al. (2015) and Harder et al. (2015) observed 4.65–5.66 Log_10_ gene copies/ml vessel fluid, whereas the concentration observed in this study was below the detection limit for the liquid phase. Furthermore, we quantified this group in the solid phase with an average concentration of 8.9 Log_10_ gene copies/g DM, which is lower than concentrations observed in grass silage-fed dairy cows (Vaidya et al., 2018). Thus, we were most probably able to quantify anaerobic fungi only during their vegetative stage in the solid phase where they physically penetrate plant material, but not during their transient motile stage that is characterized by flagellated zoospores (Gruninger et al., 2014). A possible explanation for this is the known peak in the zoospore density in ruminal fluid being directly after feed intake, i.e., 15–90 min post-feeding (Orpin, 1975, 1976, 1977), which rapidly decreases as the fungal zoospores colonize plant particles (Edwards et al., 2008). Thus, our sampling scheme could explain the low concentration of anaerobic fungi in the liquid phase, since they were quantified in pooled samples consisting of samples that were taken from the liquid phase 2, 4, 12, and 23 h after feed bag exchange.

Anaerobic fungi are important fiber degraders in the rumen (Gordon and Phillips, 1998; Paul et al., 2004). Consequently, the decrease of the anaerobic fungal concentration in the solid phase from ET to LT may partially explain the decline in fiber degradability from ET to LT previously reported (Hartinger et al., 2019b). As protozoa play a key role in the initial colonization and degradation of fibrous structures (Newbold et al., 2015), particularly hemicelluloses (Williams and Coleman, 1985), it is also possible that they too played a role. During the liquid phase sample processing, however, protozoa were separated during the first centrifugation step (Zebeli et al., 2008) and, therefore, did not allow valid assessment of the protozoal concentrations. Furthermore, Rusitec-based studies often report compromised protozoa survival in the vessels (Wallace and Newbold, 1991; Martínez et al., 2010).

Interestingly, the archaeal gene copy numbers were higher at the LT in the liquid and the solid phase, which was not expected since the daily gas production was lower at the LT than the ET (Hartinger et al., 2019b). However, it must be considered that the proportion of methane in the total gas was not determined during the *in vitro* experiment (Hartinger et al., 2019b), and the presence of methanogens may not be strictly correlated with their activity. Therefore, the methane proportion in total gas productions should be measured in future studies assessing the effect of AS pre-ensiling treatments.

During the ET, high DM AS resulted in increased archaeal numbers in the liquid phase and strong tendencies for increased abundances of bacteria and anaerobic fungi in the liquid and solid phases, respectively. Accordingly, fermentation patterns showed a strong tendency for greater gas productions and higher concentrations of propionate and isobutyrate (Hartinger et al., 2019b), which most likely originated from the tendency for higher microbial concentrations. It is speculated that the microbial concentrations could have been stimulated by the improved quality of high DM AS which resulted in less nutrient degradation during ensiling, as indicated by lower acetic acid and NPN concentrations in these AS (Hartinger et al., 2019a). However, the reduced fiber degradability of the high DM AS in the Rusitec (Hartinger et al., 2019b) contradicts this assumption as more fermentation products occurred with high DM AS, but less fiber was degraded compared to low DM AS. In this context, also the sucrose-promoted growth of anaerobic fungi was not consistent with the reduced degradability of acid detergent fiber in sucrose-treated AS (Hartinger et al., 2019b). However, it must be considered that anaerobic fungi also utilize soluble carbohydrates (Edwards et al., 2008; Gruninger et al., 2014), and likely preferred to metabolize residual sucrose instead of degrading fibrous structures of AS due to catabolite repression (Mountfort and Asher, 1983; Solomon et al., 2016).

Regarding the LT, sucrose addition increased the anaerobic fungal concentration and also high-intensity wilting showed a strong tendency to stimulate them. As high-intensity wilting increased the degradability of neutral detergent fiber (Hartinger et al., 2019b), it is speculated that this effect is at least partly explained by increased anaerobic fungal concentration. If so, then anaerobic fungi may have been more specifically involved in hemicellulose breakdown, as acid detergent fiber degradability was not affected by wilting intensity (Hartinger et al., 2019b). Concerning archaea, incubation of sucrose-treated AS led to their increased concentration in the liquid phase, but a decrease in the solid phase. The increase of liquid-associated archaea might be caused by an indirect effect since sucrose-treated AS incubation led to a higher total SCFA concentration in the vessel fluid (Hartinger et al., 2019b) and, therefore, also more hydrogen that can be used by archaea for methane production. This altogether shows that whilst sucrose addition had the largest influence of the pre-ensiling treatments on microbial concentrations, the phase was key in determining the nature of the effect.

### Microbial Community Composition

For investigating the changes in ruminal microbiota composition, we analyzed the samples from the liquid and solid phase separately. This was done as prior research already showed that clear differences exist in the microbial communities present in liquid and solid rumen content (Henderson et al., 2013; Vaidya et al., 2018). This is also in line with our findings. The number of predominant genera (i.e., those with a relative abundance > 1%) were in a similar range for both phases and time points, and compared well to the number of genera observed in ruminal *in vivo* samples (Vaidya, 2018). Since the genera *Prevotella*, *Treponema*, *Fibrobacter*, *Ruminococcus*, *Butyrivibrio*, *Pseudobutyrivibrio*, and *Methanobrevibacter* are typically present in the rumen (Bekele et al., 2011; Henderson et al., 2015; Vaidya, 2018; Vaidya et al., 2018), the predominance of these genera in the present study may indicate the existence of a core bacterial community that allows comparisons with *in vivo* assays; although the extrapolation to *in vivo* situation should be made with caution.

Since the genus *Lactobacillus* is of very low abundance in the rumen (Henderson et al., 2015), the predominance of *Lactobacillus* in the solid phase at the ET may have derived from the silages, which typically contain large quantities of this genus (Wen et al., 2017; Zheng et al., 2017). As also deducible from the RDA analysis for the solid-associated microorganisms, *Lactobacillus* was diminished at the LT, which indicated a more adapted and stable microbial community that therefore suppressed the further establishment of exogenously introduced species (Weimer et al., 2015). As the presence of *Shuttleworthia* in the rumen is increased by concentrate feeding (Plaizier et al., 2016), the predominance of this genus at the ET in the solid phase could constitute a remnant of the equilibration period, when hay plus concentrate was incubated in the Rusitec. This observation is again supported by the RDA analysis showing *Shuttleworthia* to be highly associated with the ET. *Selenomonas* harbors various proteolytic and deaminating species (Scheifinger et al., 1976; Wallace, 1985), whose predominance at both time points could be explained by the high provision of N compounds from the AS. It is also a known lactate-utilizing genus in the rumen (Mackie and Gilchrist, 1979), which further supports its high abundance. However, the RDA analysis showed no association of *Selenomonas* with the sucrose treatment that caused higher lactic acid concentrations in the silages. The genus *Megasphaera* also includes various peptidolytic, deaminating and lactate-utilizing members (Scheifinger et al., 1976; Mackie and Gilchrist, 1979; Wallace and McKain, 1991), which is likely to explain its predominance at the ET in the solid phase, but at the same time contradicts its decline toward the LT.

Although total bacterial concentrations were not affected by pre-ensiling treatments at the LT, sequencing data revealed substantial alterations in the bacterial community composition. In accordance with microbial concentration findings, the community composition data revealed that sucrose addition before ensiling had the largest effect of all the pre-ensiling treatments tested. The observed changes were more pronounced at the LT, which is most likely to be due to the longer time that the microorganisms had to adapt to the AS.

We observed a higher PD index for sucrose-treated AS at the LT, and the OTU level PCoA analysis also showed a clear separation between sucrose-treated and non-treated AS in both phases. Likewise, the RDA analysis at the genus level showed that sucrose addition was the only pre-ensiling treatment that significantly explained the observed variation in the microbial community for both phases and time points, thereby confirming the substantial impact of this pre-ensiling treatment relative to DM and wilting.

The provision of concentrate, i.e., rapidly available dietary energy, increased the microbial diversity of caprine ruminal fluid compared to ruminal fluid from solely forage-fed goats (Belanche et al., 2019b). This finding may explain the higher PD indices in microbial communities deriving from sucrose-treated AS incubations, as these silages had a lower pH as well as higher concentrations of water-soluble carbohydrates and lactic acid (Hartinger et al., 2019a), originating from sucrose metabolism of hetero- and homofermentative lactic acid bacteria during ensiling (McDonald et al., 1991). These soluble metabolites are both rapidly available energy sources for rumen microorganisms. As such, it is perhaps not surprising that the observed effects of sucrose addition were more pronounced in the liquid phase. Conversely, the crude protein and NPN proportions were higher in non-sucrose treated AS (Hartinger et al., 2019a). Since the liquid fraction is dominated by proteolytic bacteria (Plaizier et al., 2018), this may be a further reason why the liquid phase was more affected by the sucrose treatment than the solid phase.

It is important to keep in mind that besides biological causes, also the limited accessibility for sampling the solid phase in the Rusitec system is likely to have had an influence on the observed results. Due to the provision of a nylon bag with fresh feedstuff every 24 h, the microorganisms will also have relocated to the fresh AS, which in consequence may have reduced the observed impact of sucrose addition or any pre-ensiling treatment on the solid-associated population in the feed residues. Therefore, this could have promoted the stronger effect of sucrose addition observed in the liquid phase.

In contrast to RDA analysis, the PCoA analysis revealed further pre-ensiling treatment effects on microbiota beta diversity, i.e., the effects of the interactions of DM concentration and sucrose addition as well as wilting intensity and sucrose addition. These contrasting outcomes between the RDA and PCoA analysis may be partially explained by differences in data analysis approach, as PCoA analysis was performed at the OTU level based on phylogenetically-weighted pairwise distances (UniFrac), whereas RDA analysis was performed at genus level on relative abundance data. For example, OTUs within the same genus responded differently to a pre-ensiling treatment, which hence impedes the observation of an effect at the genus level. However, the pre-ensiling treatments of DM concentration and wilting intensity did not result in clear separation of the samples in the PCoA analysis. For example, this is apparent by the weak separation along the PCoA axis 1 between sucrose-treated low DM AS and sucrose-treated high DM AS (Figure 4A). Consequently, whilst statistically significant, the minor effects of these two pre-ensiling treatment effects were dwarfed by that of sucrose addition.

The second aim of this study was to investigate the effect of incubation time on the microbial community composition. Consistent with the microbial concentration findings, the sequencing data confirmed substantial alterations in the microbial community composition from ET to LT. The overall decrease of prokaryotic PD index from ET to LT may suggest a more adapted microbial community, containing bacteria that can deal better with the high provision of NPN but low availability of dietary energy (Hartinger et al., 2019a). Thus, incubation of solely AS could have caused the less diverse microbial community in both phases, especially at the LT. However, the suggested adaption is not fully reflected in the ruminal fermentation profile as both fiber and organic matter degradability decreased from ET to LT (Hartinger et al., 2019b). Martínez et al. (2010) described a reduced bacterial diversity in the Rusitec with prolonged run time. The decrease of the PD index observed from ET to LT might therefore not necessarily be due to an adaption to the AS, but a general Rusitec-derived effect.

The *Rikenellaceae* RC9 gut group is highly involved in ruminal hemicellulose degradation (Emerson and Weimer, 2017) and its predominance (i.e., highest abundant genus) at both time points in the liquid phase is not in line with the decline of fiber degradability over time. Similarly, growth of *Treponema* species is promoted by hemicelluloses and pectin (Paster and Canale-Parola, 1985; Liu et al., 2014; Emerson and Weimer, 2017) and the predominance of *Treponema* 2 (i.e., highest abundant genus) in the solid phase at both time points does again not match the declining fiber degradability with prolonged Rusitec run time.

The RDA analysis showed that several genera of *Ruminococcaceae*, including the NK4A214 group, had high relative abundances in the liquid phase samples at the ET, where we observed higher daily gas productions and fiber degradability (Hartinger et al., 2019b). Likewise, the abundance of *Ruminococcus* 1 declined in the solid phase with time, as this genus was predominant (relative abundance > 1%) at the ET, but not LT. Genera belonging to the *Ruminococcaceae* harbor major hemicellulolytic, cellulolytic and pectinolytic species (Pettipher and Latham, 1979; Nyonyo et al., 2014) and the *Ruminococcaceae* NK4A214 group was more abundant in the ruminal liquid phase of high performing dairy cows (Tong et al., 2018). Therefore, the higher presence of *Ruminococcaceae* genera, particularly the *Ruminococcaceae* NK4A214 group, might be indicative for enhanced ruminal fermentation and fiber degradation. However, only marginal information about the *Ruminococcaceae* NK4A214 group is available so far (Tong et al., 2018) and our consideration needs therefore further scientific underpinning and for the moment must be regarded with caution. Additionally, the lower presence of *Mogibacterium* and *Succiniclasticum* in the liquid phase at the LT was in line with the reduced fiber degradability since these genera promote ruminal feed degradability (Mi et al., 2018), for instance by the ability of *Mogibacterium* to form phenylacetate, which is needed by several *Ruminococcus albus* strains for cellulose degradation (Morrison et al., 1990). *Phocaeicola* was associated with the LT without sucrose addition in the solid phase, which could point to an adaptation to the pure AS diet, since this genus was shown to increase when changing sheep from hay-concentrate diets to pasture (Belanche et al., 2019a). Likewise, members of *Lachnospiraceae* are prominent pectin and hemicellulose degraders (Biddle et al., 2013), which could explain the higher presence of several *Lachnospiraceae* genera, such as the AC2044 and NK4A136 groups, in both phases at the LT. However, their high abundance is in striking contrast to the lower fiber degradability at the LT.

The RDA analysis also showed that the YAB2003 group belonging to the *Prevotellaceae* was associated with the ET in the solid phase. Since this group is involved in ruminal hemicellulose fermentation (Emerson and Weimer, 2017), its lower presence at the LT was in line with the lower fiber degradability at the LT. Consequently, although total bacterial concentrations did not decrease from ET to LT, the sequencing data shows alterations in the prokaryotic community composition that, together with the decline in anaerobic fungal concentration, may have contributed to decreased fiber degradation at the LT. Besides fibrolytic members, species of *Prevotellaceae* are also highly active at ruminal proteo- and peptidolysis (Wallace and McKain, 1991; Wallace et al., 1997; Walker et al., 2005), and the higher presence of the *Prevotellaceae* group UCG-004 at the LT in both phases could be the result of an adaption to the high NPN provision by the AS incubation.

## Conclusion

The present study demonstrates that differently produced AS influence the ruminal microbiota composition in an *in vitro* Rusitec system. Among all pre-ensiling treatments investigated, sucrose addition had the greatest effect in altering the microbial community composition in both the liquid and the solid phase. Sucrose addition increased the archaeal and anaerobic fungal concentrations in the liquid and solid phase, respectively. Likewise, PD indices were lower without sucrose addition in both phases at the LT and also PCoA analysis showed a stronger separation in community composition for the sucrose addition compared to other pre-ensiling treatments. Thus, in this study we could confirm that sucrose addition is a major driver in shaping the microbial community composition and increasing their abundances. Additionally, the time point of sampling substantially had an effect on the microbiota composition with a lower PD index at the LT and decreased concentrations of anaerobic fungi, but higher archaeal and bacterial concentrations in the solid phase. Therefore, our hypothesis of an altered microbial community composition with prolonged AS incubation could be approved. In general, all observed effects were more pronounced for the liquid than for the solid phase, most probably due to the soluble metabolites released from the incubated AS, but likely also because of the limited accessibility of the solid phase in the Rusitec system. The observed differences in the microbial composition helped to understand the alterations in the ruminal fermentation patterns, but did not fully explain them. The inclusion of metagenome, transcriptome or proteome analyses may therefore contribute to a deeper understanding of the underlying modes of action by which the pre-ensiling treatments affect the ruminal microbiota activity and fermentation.

## Data Availability Statement

The datasets generated for this study can be found in the European Nucleotide Archive. The codes and files for analysis of the prokaryotic 16S rRNA gene sequence data are deposited as a project available at https://github.com/ThHartinger/Rusitec_Microbiota. In addition, the raw sequence data is deposited in ENA under accession number PRJEB32442.

## Ethics Statement

The samples analyzed in this study were taken in the course of a previous study (Hartinger et al., 2019b). In brief, the animals used for obtaining liquid and solid ruminal content were kept according to the German Animal Welfare legislation at the Educational and Research Center Frankenforst of the Faculty of Agriculture, University of Bonn, Germany. The experimental procedures and treatments used in the previous study were in accordance with the German guidelines for animal welfare and were approved (file number 84-02.04.2017.A247) by the Animal Care Committee of the state of North Rhine-Westphalia in Germany.

## Author Contributions

TH, NG, and K-HS conceived and designed the experiment. TH performed the experiment and wrote the manuscript. TH, JE, RG, and CB analyzed the data. All authors interpreted the results, edited the manuscript, read and approved the final version of the manuscript.

## Conflict of Interest

The authors declare that the research was conducted in the absence of any commercial or financial relationships that could be construed as a potential conflict of interest.
