# Supplementary material for: Differently Pre-treated Alfalfa Silages Affect the in vitro Ruminal Microbiota Composition
Source: Front Microbiol. 2019 Dec 3;10:2761. doi: 10.3389/fmicb.2019.02761 (PMC6902091; doi:10.3389/fmicb.2019.02761)
Supplement: Supplementary file 1 [file Data_Sheet_1.docx]

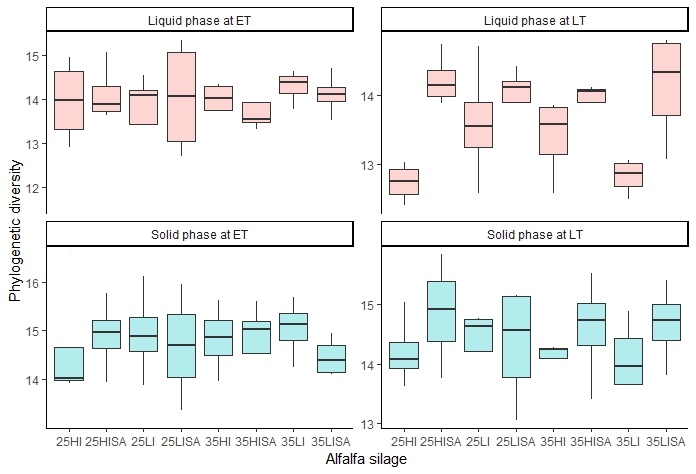


**Supplementary Figure S1.** Alpha diversity calculated with the phylogenetic diversity index for all alfalfa silages in the liquid and solid phase and for the early and late time point, respectively. The boxplots show the 25^th^, 50^th^ and 75^th^ percentiles, with whiskers showing the extremes of the data. Abbreviations indicate the treatments including different dry matter concentrations (25 or 35), wilting intensities [low (LI) or high (HI)] and sucrose addition (SA).


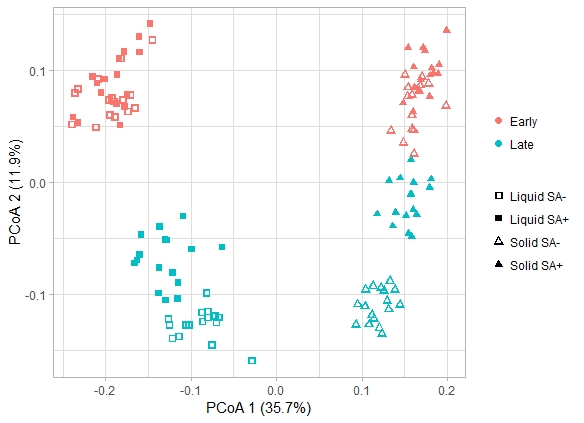


**Supplementary Figure S2.** Changes in prokaryotic community composition associated with the time point, phase, and sucrose addition visualized as a principal co-ordinate analysis (PCoA) using unweighted UniFrac distance metrics. Symbol shapes indicate the two phases, i.e., liquid and solid, from which the samples originated, colors indicate the different time points, i.e., early and late, and symbol fillings indicate the sucrose addition, i.e. with (SA+) or without (SA-). The percentage of variation explained is indicated on the respective axes.


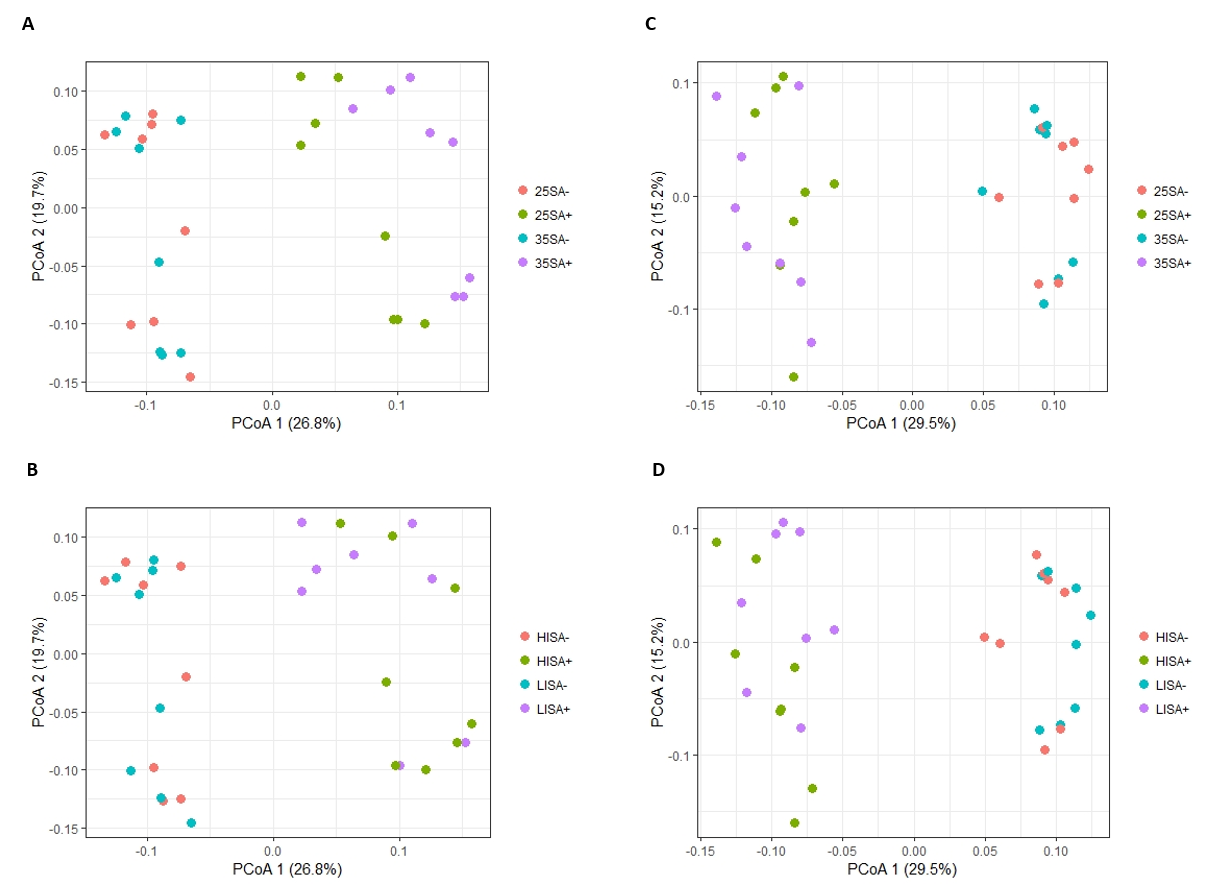


**Supplementary Figure S3.** Changes in prokaryotic community composition associated with the interactions of different pre-ensiling treatments at the late time point, visualized as a principal co-ordinate analysis (PCoA) using unweighted UniFrac distance metrics for A) treatments dry matter (DM) concentration × sucrose addition in liquid phase samples; B) wilting intensity × sucrose addition in liquid phase samples; C) DM concentration × sucrose addition in solid phase samples; D) and wilting intensity × sucrose addition in solid phase samples. Abbreviations indicate the interactions of treatments including different dry matter concentrations (25 or 35), wilting intensities (low (LI) or high (HI)) and sucrose addition (with (SA+) or without (SA-)). The percentage of variation explained is indicated on the respective axes.

**Supplementary Table S1.** Effects of dry matter concentration (DM), wilting intensity (WI) and sucrose addition (SA) on *in vitro* rumen fermentation characteristics and fiber degradability of alfalfa silages at the early time point and at the late time point. This Table represents the main fermentation and fiber degradability data presented in Hartinger et al. (2019b) *In vitro* ruminal fermentation characteristics of alfalfa silages in response to different pre-ensiling treatments. Anim. Feed Sci. Technol. 258:114306. doi: 10.1016/j.anifeedsci.2019.114306.

|  |  | Treatment^a^ | | | | | | | |  | P-values | | | | | | |  |
| --- | --- | --- | --- | --- | --- | --- | --- | --- | --- | --- | --- | --- | --- | --- | --- | --- | --- | --- |
| Time point | Item | 25HISA | 25HI | 25LISA | 25LI | 35HISA | 35HI | 35LISA | 35LI | SEM^b^ | DM | WI | SA | DM × WI | DM × SA | WI × SA |  |  |
| Early | Gas, mL/day | 1433 | 956 | 1470 | 1044 | 1533 | 1045 | 1460 | 1025 | 87.4 | 0.053 | 0.681 | **<0.001** | **0.013** | 0.786 | 0.194 |  |  |
|  | Ammonia-N, mmol/L | 30.0 | 31.1 | 29.4 | 31.7 | 29.8 | 30.1 | 32.1 | 31.7 | 0.36 | 0.186 | 0.357 | **0.006** | **0.018** | 0.196 | 0.735 |  |  |
|  | Volatile fatty acid concentration, mmol/L | | | | | | | | | | | | | | | | | |
|  | Total^c^ | 143.6 | 129.2 | 141.0 | 135.9 | 150.5 | 129.2 | 144.3 | 128.6 | 2.95 | 0.689 | 0.501 | **0.002** | 0.972 | 0.467 | 0.568 | | |
|  | Acetate | 80.4 | 76.8 | 78.8 | 80.5 | 79.8 | 78.1 | 80.5 | 76.8 | 0.57 | 0.324 | 0.296 | 0.329 | 0.497 | 0.309 | 0.741 | | |
|  | Propionate | 26.9 | 17.5 | 25.6 | 19.8 | 31.6 | 19.0 | 27.6 | 20.9 | 1.77 | **0.049** | 0.655 | **<0.001** | 0.681 | 0.369 | 0.167 | | |
|  | n-Butyrate | 18.6 | 18.9 | 18.1 | 18.1 | 22.0 | 14.8 | 19.1 | 15.6 | 0.78 | 0.386 | 0.574 | **0.004** | 0.994 | **<0.001** | 0.589 | | |
|  | Isobutyrate | 1.98 | 1.73 | 1.77 | 2.13 | 2.17 | 2.07 | 1.93 | 1.85 | 0.06 | **0.010** | 0.658 | 0.227 | **0.004** | 0.060 | 0.075 | | |
|  | Isovalerate | 7.73 | 6.81 | 7.69 | 7.51 | 7.06 | 6.93 | 6.77 | 6.05 | 0.20 | **<0.001** | 0.812 | **0.034** | **0.004** | 0.311 | 0.987 | | |
|  | Degradability, g/kg DM | | | | | | | | | | | | | | | | | |
|  | aNDFom^d^ | 437 | 387 | 345 | 323 | 326 | 356 | 299 | 323 | 5.52 | **<0.001** | **<0.001** | 0.403 | **<0.001** | **<0.001** | 0.291 | | |
|  | ADFom^e^ | 361 | 396 | 358 | 361 | 309 | 312 | 318 | 340 | 3.78 | **<0.001** | 0.984 | **0.021** | **0.007** | 0.587 | 0.606 | | |
| Late | Gas, mL/day | 1407 | 913 | 1343 | 938 | 1453 | 970 | 1490 | 945 | 92.4 | **<0.001** | 0.436 | **<0.001** | 0.997 | 0.053 | 0.424 | | |
|  | Ammonia-N, mmol/L | 32.5 | 33.9 | 34.5 | 32.8 | 31.5 | 30.6 | 31.3 | 30.8 | 0.50 | **0.012** | 0.878 | 0.560 | 0.756 | 0.572 | 0.388 | | |
|  | Volatile fatty acid concentration, mmol/L | | | | | | | | | | | | | | | | | |
|  | Total | 148.6 | 129.6 | 154.7 | 128.0 | 155.7 | 126.0 | 150.0 | 122.7 | 4.97 | 0.431 | 0.509 | **<0.001** | 0.145 | 0.266 | 0.629 | | |
|  | Acetate | 82.2 | 80.6 | 86.3 | 80.8 | 84.3 | 79.6 | 82.2 | 79.4 | 0.84 | 0.455 | 0.756 | **0.005** | 0.299 | 0.873 | 0.897 | | |
|  | Propionate | 30.1 | 13.8 | 30.9 | 16.0 | 33.1 | 18.0 | 31.4 | 17.1 | 2.91 | **0.004** | 0.822 | **<0.001** | 0.065 | 0.494 | 0.455 | | |
|  | n-Butyrate | 15.8 | 17.2 | 15.7 | 13.5 | 19.6 | 11.4 | 18.8 | 10.5 | 1.17 | 0.333 | **0.007** | **<0.001** | 0.067 | **<0.001** | **0.032** | | |
|  | Isobutyrate | 1.99 | 1.99 | 2.05 | 2.02 | 1.99 | 2.04 | 1.84 | 1.85 | 0.03 | 0.647 | 0.733 | 0.946 | 0.267 | 0.719 | 0.957 | | |
|  | Isovalerate | 11.3 | 8.49 | 11.9 | 8.58 | 9.59 | 8.23 | 8.56 | 7.29 | 0.56 | **<0.001** | 0.192 | **<0.001** | **0.038** | **<0.001** | 0.916 | | |
|  | Degradability, g/kg DM | | | | | | | | | | | | | | | | | |
|  | aNDFom | 325 | 342 | 297 | 267 | 270 | 278 | 237 | 264 | 4.31 | **<0.001** | **0.002** | 0.564 | 0.155 | 0.204 | 0.492 | | |
|  | ADFom | 234 | 285 | 269 | 275 | 204 | 235 | 223 | 247 | 3.49 | **0.003** | 0.157 | **0.016** | 0.884 | 0.973 | 0.191 | | |

^a^Treatments include different: DM concentrations, i.e. 250 g/kg DM (25) or 350 g/kg DM (35); wilting intensities, i.e. low (LI) or high (HI); and sucrose addition (SA); ^b^Standard error of the mean; ^c^Including acetate, propionate, n-butyrate, n-valerate, n-caproate, isobutyrate, and isovalerate; ^d^NDF assayed with a heat stable amylase and expressed exclusive of residual ash; ^e^ADF expressed exclusive of residual ash.

**Supplementary Table S3.** Determined p-values for all tested pre-ensiling treatments^a^ and interactions of beta diversity when applying weighted and unweighted UniFrac distance metrics.

|  |  |  | P-value | |
| --- | --- | --- | --- | --- |
| Distance metrics | Time point | Treatment | Liquid phase | Solid phase |
| Weighted UniFrac | Early | DM | 0.452 | 0.706 |
| Weighted UniFrac | Early | WI | 0.984 | 0.848 |
| Weighted UniFrac | Early | SA | 0.005 | 0.011 |
| Weighted UniFrac | Early | DM × WI | 0.945 | 0.912 |
| Weighted UniFrac | Early | DM × SA | 0.034 | 0.093 |
| Weighted UniFrac | Early | WI × SA | 0.098 | 0.185 |
| Weighted UniFrac | Late | DM | 0.031 | 0.139 |
| Weighted UniFrac | Late | WI | 0.744 | 0.561 |
| Weighted UniFrac | Late | SA | 0.001 | 0.001 |
| Weighted UniFrac | Late | DM × WI | 0.291 | 0.459 |
| Weighted UniFrac | Late | DM × SA | 0.001 | 0.001 |
| Weighted UniFrac | Late | WI × SA | 0.001 | 0.001 |
| Weighted UniFrac | Both | Time point | 0.001 | 0.001 |
| Unweighted UniFrac | Early | DM | 0.745 | 0.099 |
| Unweighted UniFrac | Early | WI | 0.940 | 0.998 |
| Unweighted UniFrac | Early | SA | 0.047 | 0.005 |
| Unweighted UniFrac | Early | DM × WI | 0.963 | 0.841 |
| Unweighted UniFrac | Early | DM × SA | 0.294 | 0.026 |
| Unweighted UniFrac | Early | WI × SA | 0.240 | 0.155 |
| Unweighted UniFrac | Late | DM | 0.259 | 0.027 |
| Unweighted UniFrac | Late | WI | 0.692 | 0.751 |
| Unweighted UniFrac | Late | SA | 0.001 | 0.001 |
| Unweighted UniFrac | Late | DM × WI | 0.788 | 0.304 |
| Unweighted UniFrac | Late | DM × SA | 0.001 | 0.001 |
| Unweighted UniFrac | Late | WI × SA | 0.001 | 0.001 |
| Unweighted UniFrac | Both | Time point | 0.001 | 0.001 |

^a^Treatments include different: dry matter (DM) concentrations, i.e. 25 or 35; wilting intensities (WI), i.e. low (LI) or high (HI); and sucrose addition (SA).
